# Neuroprotective Effects of *Carica papaya* in Experimental Models of Neurodegeneration: A Comprehensive Review

**DOI:** 10.1002/fsn3.72507

**Published:** 2026-10-21

**Authors:** Rakib Hossan, Samy Selim, Imam Hossen Rakib, Md Abu Sayeed, Anike Chakrabarty, Md. Arif Hossain, Emon Mia, Dristy Debnath, Asmaul Husna Bristy, Md. Sakib Al Hasan

**Affiliations:** ^1^ Department of Pharmacy Gopalganj Science and Technology University Gopalganj Bangladesh; ^2^ Department of Clinical Laboratory Sciences, College of Applied Medical Sciences Jouf University Sakaka Saudi Arabia; ^3^ Alor Dishari Research Organization Dhaka Bangladesh; ^4^ Department of Pharmacy Mawlana Bhashani Science and Technology University Santosh, Tangail Bangladesh; ^5^ Department of Biotechnology, Faculty of Engineering and Science University of Greenwich Kent UK; ^6^ Department of Pharmacy University of Asia Pacific Dhaka Bangladesh

**Keywords:** Alzheimer's disease, *Carica papaya*, neuroinflammation, neuroprotective, oxidative stress

## Abstract

*Carica papaya* ( *C. papaya* ) has long been used in traditional medicine, and experimental studies have reported neuroprotective effects across several models; however, the available evidence is distributed across different plant parts, preparations, and experimental systems. This review synthesizes existing evidence from in vitro and in vivo studies assessing the neuroprotective, antioxidant, anti‐inflammatory, and neuromodulatory effects of *C. papaya* extracts derived from seeds, leaves, pulp, fruit, and fermented preparations. A comprehensive literature search of PubMed, Scopus, Web of Science, Google Scholar, and ScienceDirect identified relevant studies across multiple experimental models through 22 September 2026. Findings show that in Alzheimer's disease (AD) models, aluminum‐induced neurotoxicity, D‐galactose‐induced brain aging, 6‐hydroxydopamine (6‐OHDA)‐mediated oxidative damage, and chemically induced neurotoxicity (cyclophosphamide, acrylamide, and fluoride), *C. papaya* preparations were associated with improvements in selected cognitive, behavioral, biochemical, and oxidative‐stress‐related outcomes across the included experimental models. Toxicity studies generally reported no mortality within the tested dose ranges; however, behavioral, biochemical, and mild histopathological alterations were observed in some studies, particularly at higher doses, indicating that safety may vary according to extract type, dose, and duration of exposure. Overall, the available preclinical evidence suggests that *C. papaya* preparations may affect multiple pathways relevant to oxidative‐stress‐associated neurodegeneration. However, further mechanistic studies, bioactive compound isolation, pharmacokinetic assessments, and well‐designed clinical trials are essential to support its future therapeutic development.

## Graphical abstract

*Carica papaya* preparations have produced changes in antioxidant, Nrf2‐related, cholinergic, amyloid‐associated, inflammatory, apoptotic, neuronal, and behavioral endpoints in preclinical models. The evidence is model‐ and preparation‐specific, and clinical efficacy has not been established.

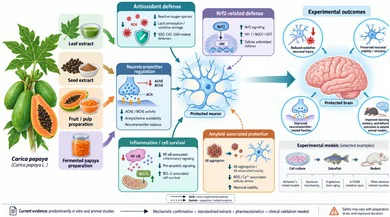

**Abbreviations**
6‐OHDA: 6‐Hydroxydopamine
ABTS: 2, 2′‐Azino‐bis(3‐ethylbenzothiazoline‐6‐sulfonic acid)
ACh: acetylcholine
AChE: acetylcholinesterase
AD: Alzheimer's disease
ALP: alkaline phosphatase
ALT: alanine aminotransferase
APP: amyloid precursor protein
ARE: antioxidant response element
AST: aspartate aminotransferase
ATP: adenosine triphosphate
Aβ: amyloid‐beta
Bax: Bcl‐2‐associated X protein
BChE: butyrylcholinesterase
Bcl‐2: B‐cell lymphoma 2
bw: body weight
CAT: catalase
CPLE:
*Carica papaya* leaf extract
CPSC:
*Carica papaya* leaf scCO_2_ extract
DPPH: 2, 2‐Diphenyl‐1‐picrylhydrazyl
FPP: fermented papaya preparation
FRAP: ferric reducing antioxidant power
G6PDH: glucose‐6‐phosphate dehydrogenase
GABA: γ‐aminobutyric acid
GPx/GPX: glutathione peroxidase
GR: glutathione reductase
GSH: reduced glutathione
GST: glutathione S‐transferase
HD: Huntington's disease
HO‐1: heme oxygenase‐1
HOCl: hypochlorous acid
IC_50_
: half‐maximal inhibitory concentration
IgG: immunoglobulin G
IgM: immunoglobulin M
IL‐6: interleukin‐6
IL‐8: interleukin‐8
i.p.: intraperitoneal
IROS: intracellular reactive oxygen species
JNK: c‐Jun N‐terminal kinase
LC–MS: liquid chromatography‐mass spectrometry
LD_50_
: median lethal dose
MCV: mean corpuscular volume
MDA: malondialdehyde
*n*
: number of animals/experiments
NDs: neurodegenerative disorders
NF‐κB: nuclear factor kappa‐light‐chain‐enhancer of activated B cells
NO: nitric oxide
NQO1: NAD(P)H quinone oxidoreductase 1
Nrf2: nuclear factor erythroid 2‐related factor 2
ORAC: oxygen radical absorbance capacity
PD: Parkinson's disease
PEECPS: petroleum ether extract of *Carica papaya* seeds
R/A: route/administration
ROS: reactive oxygen species
scCO_2_
: supercritical carbon dioxide
SH‐SY5Y: human neuroblastoma cell line
SOD: superoxide dismutase
TBARS: thiobarbituric acid‐reactive substances
TNF‐α: tumor necrosis factor‐alpha
XO: xanthine oxidase

## 1 Introduction

Neurodegenerative disorders (NDs) are a group of progressive conditions characterized by the gradual loss of neuronal structure and function, leading to cognitive, motor, and behavioral impairments. Common examples include AD, Parkinson's disease (PD), Huntington's disease (HD), and stroke‐related neurodegeneration (Rosenberg 2017). These disorders pose a significant global health challenge, with AD alone affecting over 50 million people worldwide and projected to triple by 2050 due to aging populations (Chen et al. 2024). The public health burden is substantial, with NDs contributing to significant morbidity, mortality, and economic costs, including an estimated $1 trillion annually for AD‐related care (Prince et al. 2015).

The pathophysiology of NDs is complex and multifactorial, involving oxidative stress, neuroinflammation, mitochondrial dysfunction, and protein aggregation. Oxidative stress arises from an imbalance between reactive oxygen species (ROS) production and antioxidant defenses, leading to neuronal damage (Halliwell 2012). Neuroinflammation, driven by activated microglia and pro‐inflammatory cytokines, exacerbates neuronal loss (Heneka et al. 2015). Mitochondrial dysfunction impairs energy production and increases ROS, contributing to neurodegeneration (Lin and Beal 2006). Protein aggregation, such as Aβ plaques in AD or alpha‐synuclein in PD, disrupts cellular homeostasis and triggers neuronal death (Singh and Dey 2025). Current treatments for neurodegenerative disorders are disease‐specific and often provide symptomatic benefit, while their effects on disease progression vary among disorders and therapies. For instance, cholinesterase inhibitors for AD or levodopa for PD provide temporary benefits but fail to halt disease progression and are often associated with adverse effects, such as nausea, hallucinations, and cognitive disturbances (Olanow et al. 2009).

A natural product is a chemical compound or substance that is produced by living organisms, such as plants, animals, microorganisms, or marine organisms (Bartkowiak‐Wieczorek and Mądry 2024). Natural products often possess pharmacological or biological activities (Les et al. 2024). Natural products, particularly medicinal plants, have a long history of use in traditional medical systems, including Ayurveda and Traditional Chinese Medicine (Howes and Houghton 2003). Plants such as *Ginkgo biloba* , *Curcuma longa* , and *Panax ginseng* have been used for centuries to enhance cognitive function and mitigate neurological symptoms. Some phytochemicals have been reported to modulate multiple molecular pathways that may be relevant to the multifactorial mechanisms of NDs.

*C. papaya* L., commonly known as papaya, is an herbaceous perennial plant belonging to the family Caricaceae, within the order Brassicales (Yogiraj et al. 2014). It features a single, semi‐woody, hollow stem reaching heights of 2–10 m, crowned with large, palmately lobed leaves supported by long petioles containing milky latex vessels (Saran et al. 2016). The plant is typically dioecious, bearing male or female flowers, though hermaphroditic forms exist, producing spherical to cylindrical fruits that can weigh up to 9–11.5 kg (Fuentes and Santamaría 2014). *C. papaya* has a long history of use in traditional medicine for a range of conditions, including fever, asthma, gastrointestinal disorders, and inflammatory conditions (Krishna et al. 2008). Experimental studies have also demonstrated antioxidant and anti‐inflammatory activities of different *C. papaya* preparations. For example, *C. papaya* ‐containing plant extracts have been shown to reduce TNF‐α production in immune‐cell models (Sagnia et al. 2014), while papaya seed extracts exhibit free‐radical‐scavenging and antioxidant activities (Zhou et al. 2011). In addition, *C. papaya* exhibits broad anti‐inflammatory and immunomodulatory properties, with reported modulation of pro‐inflammatory cytokines and NF‐κB signaling, suggesting potential relevance to neuroinflammatory processes; however, direct evidence in neurological models remains limited (Pandey et al. 2016). These findings provide evidence of antioxidant and immunomodulatory activities of selected *C. papaya* preparations. Oxidative stress and neuroinflammation are established components of the pathophysiology of several NDs (Uttara et al. 2009). Therefore, the potential neurological relevance of *C. papaya* should be evaluated primarily from studies that directly assess neuronal, neurochemical, neuropathological, or neurobehavioral outcomes.

Despite these promising observations, evidence regarding the neuroprotective effects of *C. papaya* remains dispersed across different experimental models, plant parts, extract types, and proposed mechanistic pathways. In addition, the specific bioactive compounds responsible for these effects, their molecular targets, and their translational relevance to human neurological disorders remain incompletely characterized. Therefore, this review aims to critically analyze and synthesize the available evidence on the neuroprotective properties of *C. papaya* across diverse experimental models. Specifically, this review: (i) evaluates evidence from in vitro and in vivo studies assessing the neuroprotective, antioxidant, anti‐inflammatory, and neuromodulatory effects of different *C. papaya* preparations, including seeds, leaves, pulp, fruit, and fermented preparations; (ii) compares findings across experimental models of Alzheimer's disease, aluminum‐induced neurotoxicity, D‐galactose‐induced brain aging, 6‐hydroxydopamine (6‐OHDA)‐mediated oxidative damage, and chemically induced neurotoxicity; (iii) identifies current knowledge gaps concerning bioactive compound characterization, mechanistic pathways, and clinical translation; (iv) summarizes available evidence from acute, subchronic, and chronic toxicity studies; and (v) discusses priorities for future research, including extract standardization, pharmacokinetic characterization, and clinical investigation. By integrating findings across these experimental settings, this review provides a comparative overview of the current evidence and identifies areas requiring further investigation before the therapeutic relevance of *C. papaya* in ND disorders can be established.

## 2 Methodology

### 2.1 Search Strategy

A literature search was conducted in PubMed, Scopus, Web of Science, Google Scholar, and ScienceDirect from database inception through 22 September 2026. Search terms included combinations of “ *Carica papaya* ” or “papaya extract” with “neuroprotection,” “neuroprotective activity,” “antioxidant,” “phytochemicals,” “neurodegenerative disease,” “toxicology,” “clinical trial,” and terms describing in vitro or in vivo neurological studies. Reference lists of eligible articles were also screened for additional studies.

### 2.2 Inclusion Criteria

Studies were included if they met the following criteria:

- Investigated a *C. papaya* ‐derived extract or preparation.
- Reported outcomes related to neuroprotective, antioxidant, anti‐inflammatory, or neuromodulatory effects.
- Used in vitro or in vivo experimental designs or comprised toxicological or clinical investigations.
- Published in peer‐reviewed journals and written in English.
- Provided sufficient methodological details and clearly defined neuroprotective outcomes.

### 2.3 Exclusion Criteria

Studies were excluded based on the following criteria:

- Articles not directly related to neuroprotection or neurological parameters.
- Reviews, conference abstracts, book chapters, and non–peer‐reviewed sources.
- Studies focusing solely on nutritional, agricultural, or non‐pharmacological properties of papaya.
- Papers with insufficient data, unclear methodology, or inaccessible full text.
- Non‐English publications.

### 2.4 Study Selection Process

All identified records were compiled and duplicates were removed before screening. Titles and abstracts were screened against the predefined eligibility criteria, followed by full‐text assessment. Studies evaluating direct neuronal, neurochemical, neuropathological, or neurobehavioral outcomes were treated as direct neurological evidence. Studies that measured only systemic antioxidant, inflammatory, or related mechanistic endpoints were retained as indirect supporting evidence when relevant to pathways involved in neurodegeneration. Additional studies were identified through reference‐list screening. Figure 1 summarizes the identification, screening, eligibility assessment, and final study inclusion using a Preferred Reporting Items for Systematic reviews and Meta‐Analyses (PRISMA)‐style flow diagram.

**FIGURE 1:**
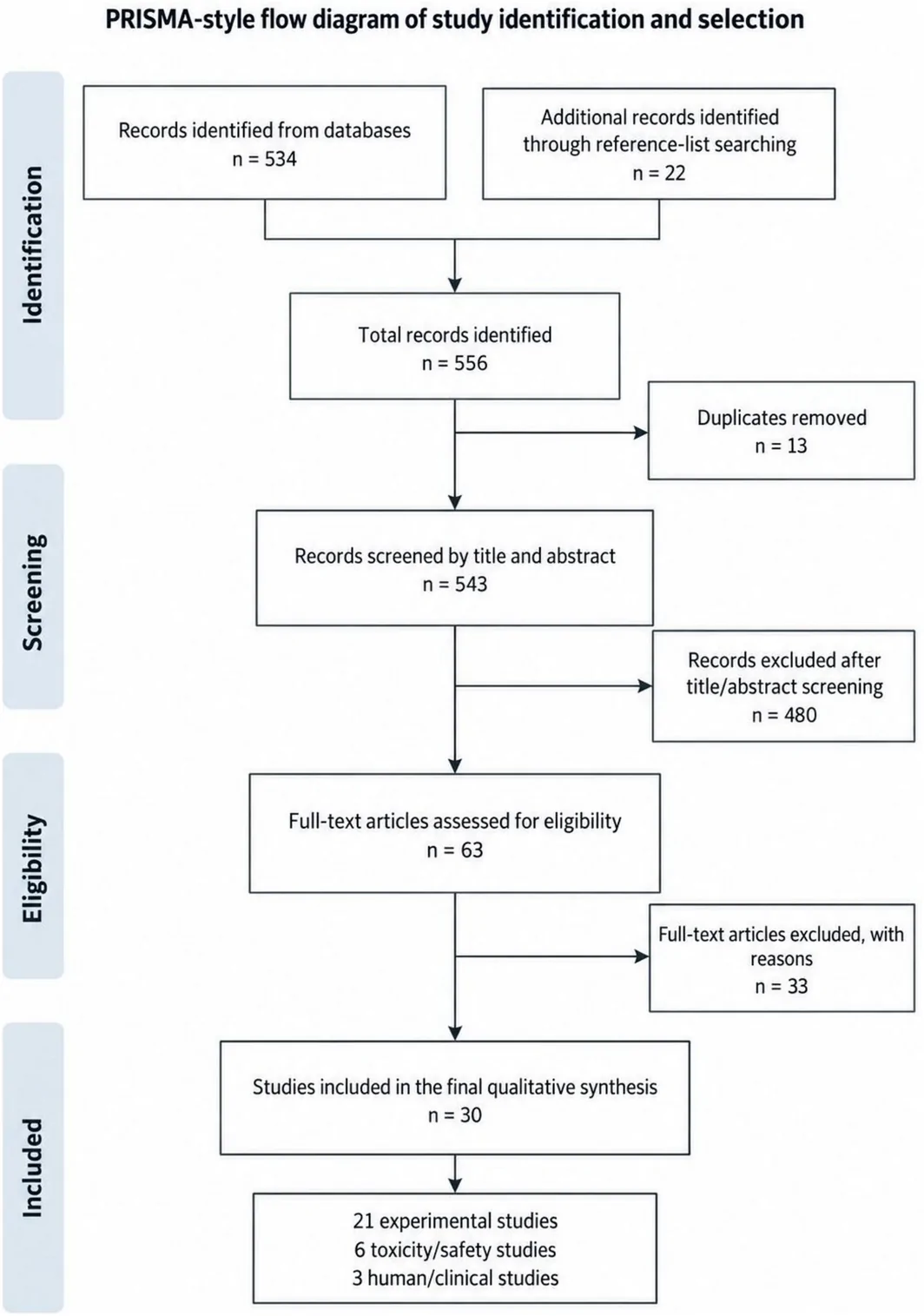
PRISMA‐style flow diagram summarizing the literature identification, screening, eligibility assessment, and final inclusion of studies in this comprehensive review.

## 3 Results and Discussion

### 3.1 Phytochemical Constituents and Bioactive Compounds of *Carica papaya*

*C. papaya* contains chemically diverse constituents distributed unevenly among the fruit, peel, leaves, seeds, latex, and roots. The major phytochemical and nutritional constituents reported in the literature include phenolic acids, flavonoids, carotenoids, glucosinolates, cyanogenic glycosides, alkaloids, tannins, saponins, terpenoids, vitamins, minerals, and proteolytic enzymes (Khatun et al. 2026; Cao et al. 2024). Another study found that papaya fruit contains phenolic acids and flavonoids, including caffeic acid, protocatechuic acid derivatives, gallic acid derivatives, quercetin, rutin, kaempferol, myricetin, and isorhamnetin. In liquid chromatography–mass spectrometry (LC–MS) analyses, several of these compounds were detected in the peel, whereas the flesh contained mainly caffeic acid and conjugated protocatechuic and gallic acid derivatives (Rivera‐Pastrana et al. 2010). However, the pulp contains carotenoids, predominantly lycopene, followed by β‐cryptoxanthin and β‐carotene, which contribute to its antioxidant potential (Cao et al. 2024). A separate study reported that papaya leaves contain a complex mixture of phenylpropanoids and flavonoids. Metabolomic analysis identified caffeic, cinnamic, chlorogenic, quinic, coumaric, vanillic, and protocatechuic acids, together with naringenin, hesperidin, rutin, and kaempferol (Gogna et al. 2015). A previous study reported that papaya seeds are rich in benzyl glucosinolate (glucotropaeolin), which can yield benzyl isothiocyanate upon myrosinase‐mediated hydrolysis; these compounds were largely absent from the pulp and showed in vitro antioxidant and pro‐apoptotic effects (Nakamura et al. 2007). Overall, *C. papaya* exhibits a diverse and differentially distributed phytochemical profile, with its bioactive constituents. These studies indicate that the phytochemical composition of *C. papaya* varies considerably among plant parts.

### 3.2 In Vitro Antioxidant and Enzyme Inhibition Studies of *Carica papaya*

*C. papaya* exhibits antioxidant, cholinesterase‐inhibitory, and cytoprotective activities that may be relevant to neurological disorders, particularly AD and oxidative‐stress‐associated neurodegeneration. The methanolic leaf extract showed 2,2‐Diphenyl‐1‐picrylhydrazyl (DPPH) and 2,2′‐Azino‐bis(3‐ethylbenzothiazoline‐6‐sulfonic acid) (ABTS) radical‐scavenging activities with IC_50_ values of 59.27 and 39.84 μg/mL, respectively, together with acetylcholinesterase (AChE) inhibitory activity. These findings represent distinct antioxidant and cholinesterase‐related activities (Adetuyi et al. 2024). Similarly, an alkaloid‐enriched leaf extract produced 75.9% butyrylcholinesterase (BChE) inhibition, suggesting a potential reduction in acetylcholine (ACh) hydrolysis and consequent enhancement of cholinergic neurotransmission, a mechanism relevant to AD (Khaw et al. 2020). Aqueous *C. papaya* leaf extract exhibited 75.68% xanthine oxidase (XO) inhibition in an assay primarily relevant to hyperuricemia and gout (Azmi et al. 2012). Although XO‐derived reactive oxygen species can contribute to oxidative stress, this study did not evaluate neuronal cells, brain tissue, neurobehavioral outcomes, or ND disease models. Therefore, its findings should be considered indirect mechanistic evidence rather than direct evidence of neuroprotection. Different leaf extracts obtained by supercritical‐fluid and conventional extraction methods demonstrated antioxidant and cytoprotective properties, supporting their ability to reduce oxidative cellular injury (Khor et al. 2021). Similarly, aqueous unripe fruit extract demonstrated substantial antioxidant activity and protected against oxidative stress in a non‐neuronal endothelial model (Jarisarapurin et al. 2019). These antioxidant and cytoprotective effects may be mechanistically relevant to conditions involving oxidative stress; however, they do not directly establish neuroprotective activity.

In contrast, fermented papaya preparation (FPP) provides direct experimental evidence relevant to neuroprotection. In APP/APPsw‐overexpressing SH‐SY5Y neuroblastoma cells, FPP attenuated copper‐associated Aβ neurotoxicity, increased SOD activity, and reduced intracellular Ca^2+^, ROS, NO, and Aβ–Cu^2+^‐mediated cellular injury (Zhang et al. 2006) (Table 1 and Figure 2). Thus, evidence from neuronal models should be distinguished from antioxidant or cytoprotective findings obtained in non‐neuronal experimental systems.

**TABLE 1:** In vitro antioxidant and enzyme‐inhibitory activities of *C. papaya* extracts and their direct or indirect relevance to neuroprotection.

Plant part	Extract type	Assay/Target enzyme	Key result/IC_50_	Mechanism	Evidence level	References
Leaves	Methanol extract	DPPH radical scavenging	59.27 μg/mL	↑Radical‐scavenging capacity	Indirect	Adetuyi et al. (2024)
		ABTS radical scavenging	39.84 μg/mL	**↑**Antioxidant activity		
		AChE inhibition	60.95 μg/mL	↓AChE inhibition		
	Alkaloid‐enriched leaf extract	Ellman enzyme inhibition assay	75.9% BChE inhibition	**↓**BChE		Khaw et al. (2020)
Mature leaves	Aqueous (distilled‐water) leaf extract	XO inhibition	75.68% inhibition	↓XO, uric acid formation, oxidative stress associated with XO activity		Azmi et al. (2012)
Leaves	scCO_2_ extract (CPSC)	DPPH radical‐scavenging assay	**—**	↑Radical‐scavenging capacity ↓ROS		Khor et al. (2021)
Unripe fruit	Aqueous extract of unripe fruit	FRAP, ORAC; ROS‐scavenging assays	FRAP = 26.89 μmol/g; ORAC (trolox equivalent) = 56.03 μmol/g	↑Antioxidant capacity ↓ROS		Jarisarapurin et al. (2019)
		H_2_O_2_ scavenging	196.39 μg/mL	↓H_2_O_2_		
		OH^•^ scavenging	280.17 μg/mL	↓Hydroxyl radicals		
		Superoxide (O_2_^•–^) scavenging	846.53 μg/mL	↓Superoxide radicals		
		HOCl scavenging	1316.13 μg/mL	**↓**HOCl		
Fruit	Fermented papaya preparation (FPP/PS‐501)	APP/APPsw‐overexpressing SH‐SY5Y neuroblastoma cells, copper‐induced Aβ neurotoxicity	—	↑SOD ↓intracellular Ca^2+^, ROS, NO, Aβ–Cu^2+^‐mediated neurotoxicity	Direct neuronal evidence	Zhang et al. (2006)

Abbreviations: ↑, increase/upregulation/stimulation; ↓, decrease/downregulation/Inhibition; ABTS, 2,2′‐Azino‐bis(3‐ethylbenzothiazoline‐6‐sulfonic acid); APP, amyloid precursor protein; Aβ, amyloid‐β; BChE, butyrylcholinesterase; CPSC, *Carica papaya* leaf scCO2 extract; DPPH, 2,2‐Diphenyl‐1‐picrylhydrazyl; FRAP, ferric reducing antioxidant power; HOCl, hypochlorous acid; IC_50_, half‐maximal inhibitory concentration; NO, nitric oxide; ORAC, oxygen radical absorbance capacity; ROS, reactive oxygen species; scCO_2_, supercritical carbon dioxide; SOD, superoxide dismutase; XO, xanthine oxidase.

**FIGURE 2:**
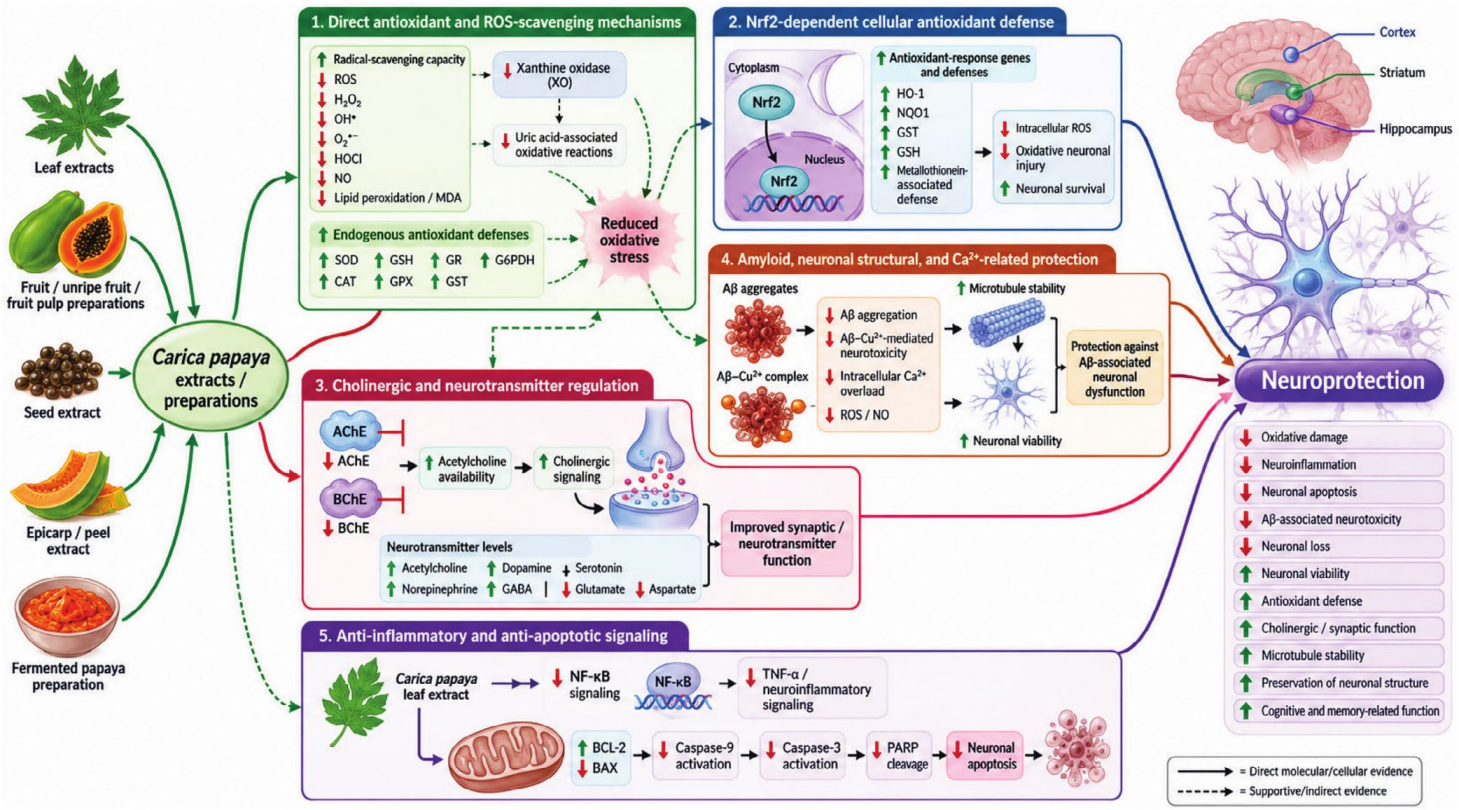
Proposed neuroprotective mechanisms of *C. papaya* extracts and preparations [Reported actions include antioxidant effects through ROS reduction, inhibition of lipid peroxidation and XO activity, enhancement of endogenous antioxidants, and activation of Nrf2‐related pathways. Neuroprotective effects also involve AChE/BChE inhibition, modulation of neurotransmitters, reduced Aβ aggregation and Aβ–Cu^2+^ neurotoxicity, attenuation of Ca^2+^ and ROS/NO stress, suppression of NF‐κB/TNF‐α signaling, and regulation of BCL‐2/BAX, caspases, and PARP. These mechanisms may reduce oxidative stress, neuroinflammation, apoptosis, and neuronal loss while supporting neuronal viability, synaptic function, and cognition. Solid arrows indicate directly demonstrated mechanisms; dashed arrows indicate supportive or indirect evidence. AChE, acetylcholinesterase; Aβ, amyloid‐β; BAX, BCL‐2‐associated X protein; BChE, butyrylcholinesterase; BCL‐2, B‐cell lymphoma 2; NF‐κB, nuclear factor kappa B; Nrf2, nuclear factor erythroid 2‐related factor 2; PARP, Poly(ADP‐ribose) polymerase; ROS, reactive oxygen species; TNF‐α, tumor necrosis factor‐α; XO, xanthine oxidase].

Collectively, these findings support antioxidant, enzyme‐inhibitory, and cytoprotective activities of *C. papaya* . However, findings obtained from non‐neuronal assays or models should be regarded as indirect mechanistic evidence, whereas neuroprotective activity is more directly supported by studies using neuronal cells or neurological experimental models.

### 3.3 Alzheimer's Disease Models

#### 3.3.1 Alzheimer's Disease in Zebrafish

In AD, excessive oxidative stress and ROS damage neurons, disrupt synaptic signaling, and impair neurotransmitters required for learning and memory (Tönnies and Trushina 2017), potentially leading to increased anxiety, reduced exploratory behavior, and impaired spatial working memory, as reflected by decreased spontaneous alternation between goal arms (d'Isa et al. 2021). In experimental models, interventions with antioxidant or neuroprotective activity may reduce oxidative damage and improve selected neuronal or behavioral outcomes (Teleanu et al. 2019). As a result, animals show increased entries into the green arm/light chamber and decreased entries into the red arm/dark chamber, indicating improved cognition and reduced anxiety. Shetty and Bharathi (2024) reported that seed extract (50–100 mg/kg) showed neuroprotective activity in an in vivo zebrafish model of AD. After treatment, fish exhibited improved cognitive and behavioral performance, demonstrated by increased time spent and total entries into the green arm of the T‐maze and the light chamber. Conversely, the extract significantly decreased the time spent and total number of entries into the red arm of the T‐maze and the dark chamber, indicating reduced anxiety, better spatial memory, and protection against Alzheimer's‐related neuronal damage (Shetty and Bharathi 2024). Histological and AChE findings were also reported. These behavioral findings were accompanied by biochemical and histological outcomes. However, these results are limited to the measured behavioral, biochemical, and histological endpoints in this zebrafish model and should not be interpreted as evidence of clinical efficacy in Alzheimer's disease.

#### 3.3.2 Alzheimer's Disease Using PC12 Cells

Aβ aggregation is a major pathological feature of AD and is associated with neuronal dysfunction (Bharadwaj et al. 2009). When Aβ peptides accumulate and form oligomers and plaques, they trigger excessive oxidative stress, damage neuronal membranes, and disrupt synaptic communication (Butterfield 2002). These aggregates also induce inflammation and mitochondrial dysfunction, leading to progressive neuronal death. As a result, memory, learning, and overall cognitive functions decline, contributing to pathological processes associated with AD (Sehar et al. 2022). The methanolic extract of papaya leaves was reported to confer a concentration‐dependent neuroprotective effect against anti‐NGF‐induced cytotoxicity in PC12‐derived neurons. This protection is mechanistically attributed to the simultaneous inhibition of pathogenic protein aggregation and preservation of cytoskeletal microtubule integrity, thereby enhancing overall neuronal viability (Savla et al. 2017). By suppressing Aβ buildup and protecting neuronal integrity, the extract reduced the measured oxidative‐stress‐ and neuronal‐injury‐related endpoints in this cellular model. However, these findings describe cellular and protein‐aggregation endpoints in vitro and do not establish therapeutic effects in patients with Alzheimer's disease.

### 3.4 Aluminum‐Induced Neurotoxicity Models

#### 3.4.1 Aluminum‐Induced Cognitive Dysfunction in Wistar Rats and Cell Models

Aluminum‐induced cognitive dysfunction occurs because aluminum accumulates in the brain and disrupts normal neuronal function (Renke et al. 2023). It promotes excessive generation of ROS, which leads to oxidative damage of lipids, proteins, and neuronal membranes (Wu et al. 2012). Aluminum also interferes with cholinergic neurotransmission and impairs antioxidant enzymes, weakening the brain's defense system (Rahman and Banu 2024). Together, these effects cause memory impairment, reduced learning ability, and progressive neuronal injury. Bindhu and Vijayalakshmi (2019) reported that the leaf extract exhibits neuroprotective activity in male Wistar rats with aluminum‐induced cognitive dysfunction and oxidative damage. In their in vivo study (*n* = 6 per group), rats received 200–400 mg/kg of the *C. papaya* leaf extract orally, which significantly increased key antioxidant markers such as SOD, catalase (CAT), glutathione reductase (GR), lipid peroxidation control, and ACh levels (Bindhu and Vijayalakshmi 2019). However, the limited sample size reduces the statistical precision and generalizability of these findings, and confirmation in larger, adequately powered studies is required. Another study found that Papaya epicarp extract (30 μg/mL) protected SH‐SY5Y neuronal cells against aluminum‐induced neurotoxicity by reducing oxidative damage and enhancing neuroprotection (Waly et al. 2012). However, these findings apply to the measured experimental endpoints in the respective models. In addition, these biochemical changes are consistent with reduced oxidative injury in the experimental model by counteracting oxidative stress, restoring antioxidant balance, and supporting cognitive function.

#### 3.4.2 Aluminum Lactate‐Induced Alzheimer‐Like Disease

Aluminum‐induced Alzheimer's‐like disease occurs when aluminum accumulates in the brain, where it disrupts neuronal function and promotes neurotoxicity (Savory et al. 2006). Aluminum enhances the production of ROS, causing oxidative stress that damages lipids, proteins, and DNA in neurons (Wu et al. 2012). It also promotes Aβ aggregation and tau hyperphosphorylation, leading to plaque and tangle formation. Additionally, aluminum impairs cholinergic neurotransmission, triggers neuroinflammation, and inhibits antioxidant defense systems, collectively resulting in cognitive deficits, memory loss, and Alzheimer's‐like neurodegeneration (Tyczyńska et al. 2024). Al‐Okbi et al. (2017) investigated *C. papaya* extracts in male albino rats (*n* = 6) with aluminum lactate‐induced Alzheimer‐like neurotoxicity. Aluminum exposure increased oxidative stress, inflammation, and cholinesterase activity, as shown by elevated brain and plasma malondialdehyde (MDA), TNF‐α, nitrite, plasma cholinesterase, and brain AChE. Treatment with 250 mg/kg of the papaya leaf, fruit, and seed extracts significantly improved several of these alterations. Papaya leaf extract produced the strongest reduction in brain AChE activity, while papaya fruit extract reduced both brain AChE and lipid peroxidation. The petroleum ether extract of papaya seeds showed the greatest reduction in brain MDA, indicating a strong antioxidant effect. Papaya extracts also reduced inflammatory markers and improved antioxidant status. These findings support the neuroprotective potential of *C. papaya* through antioxidant, anti‐inflammatory, and anticholinesterase mechanisms. However, the study assessed mainly biochemical markers and did not evaluate cognition, amyloid‐*β*, tau pathology, or detailed histological changes. Therefore, the results provide experimental support for *C. papaya* in aluminum‐induced neurotoxicity but require further validation in more disease‐specific models of Alzheimer's disease (Al‐Okbi et al. 2017).

### 3.5 D‐Galactose‐Induced Brain Aging

D‐galactose induced brain aging occurs through excessive production of ROS and the accumulation of advanced glycation end‐products, which collectively trigger oxidative damage to neuronal cells (Liu et al. 2013). These disturbances further lead to mitochondrial dysfunction and chronic neuroinflammation, impairing energy metabolism and promoting inflammatory cytokine release (Younis et al. 2024). As a result, neurotransmitter balance is disrupted and neuronal apoptosis increases, causing deficits in learning, memory, and behavior. Naz et al. (2022) investigated the neuroprotective effects of seed and pulp extract in a D‐galactose–induced brain‐aging model using Albino Wistar rats (*n* = 5). The extract was administered intraperitoneally at a dose of 150 mg/kg, and it significantly improved cognitive and behavioral outcomes. Treated rats showed enhanced cognitive learning ability, memory performance, muscular strength, and spatial memory, along with elevated levels of γ‐aminobutyric acid (GABA) and key antioxidant enzymes such as SOD, glutathione peroxidase (GPX), and CAT (Naz et al. 2022). These findings suggest potential neuroprotective effects against D‐galactose‐induced brain aging. However, the study included only five animals, and therefore the observed biochemical and behavioral effects should be interpreted cautiously and require replication in larger, adequately powered experimental studies.

### 3.6 General Oxidative Damage Models

6‐OHDA induces oxidative stress by entering dopaminergic and noradrenergic neurons via catecholamine transporters (Dooley et al. 2012). Inside the neuron, it undergoes auto‐oxidation and enzymatic metabolism, generating excessive ROS such as superoxide anions, hydrogen peroxide, and hydroxyl radicals (Bayo‐Olugbami et al. 2022). These ROS damage cellular lipids, proteins, and DNA, impair mitochondrial function, and trigger apoptotic pathways. The resulting oxidative stress leads to selective neuronal death, especially in dopaminergic pathways, contributing to neurodegeneration (Guo et al. 2018). Murakami et al. (2018) demonstrated that FPP enhanced the antioxidant defense capacity of astrocytes through nuclear factor erythroid 2‐related factor 2 (Nrf2) activation. FPP increased nuclear translocation of Nrf2 and upregulated Nrf2‐regulated cytoprotective molecules, including NAD(P)H quinone oxidoreductase 1 (NQO1), glutathione S‐transferase (GST), and heme oxygenase‐1 (HO‐1), while also increasing GSH levels and metallothionein‐expressing astrocytes. In neuronal models, FPP pretreatment suppressed 6‐OHDA‐induced dopaminergic neuronal loss, indicating protection against neurotoxin‐induced neuronal damage rather than a reduction in 6‐OHDA itself. Furthermore, a 2‐week oral administration of FPP activated Nrf2 and increased striatal GSH levels in mice. Thus, the neuroprotective effect was associated with Nrf2‐mediated enhancement of antioxidant and detoxification defenses, which attenuated 6‐OHDA‐induced dopaminergic neurodegeneration (Murakami et al. 2018). These findings are consistent with a neuroprotective effect of FPP in the evaluated experimental systems by enhancing endogenous antioxidant defenses, reducing oxidative stress, and supporting overall neuronal survival and function. Moreover, Oloyede et al. (2011) reported antioxidative effects of the fruit extract in albino mice (*n* = 8) subjected to oxidative damage. Administered orally at 100 mg/kg, the extract significantly increased key antioxidant enzymes and protective markers, including GST, GPX, GR, and glucose‐6‐phosphate dehydrogenase (G6PDH) (Oloyede et al. 2011). However, this does not represent a direct neuroprotective effect; the observed antioxidant activity may indirectly contribute to neuroprotection by reducing oxidative stress, which is implicated in neuronal injury and neurodegeneration. In a separate study, papaya leaf extract protected H_2_O_2_‐stressed SH‐SY5Y cells by reducing ROS and NF‐κB signaling while increasing Nrf2/HO‐1 expression and anti‐apoptotic BCL‐2 (Jeon et al. 2025). In addition, Papaya epicarp extract also reduced H_2_O_2_‐induced oxidative injury and improved cell‐viability‐related outcomes in SH‐SY5Y cells (Guizani et al. 2011). Zeweil et al. (2026) evaluated papaya pulp juice as an adjunct to semaglutide in a streptozotocin‐induced rat model of diabetic encephalopathy. Rats received semaglutide (0.3 mg/kg, s.c.) combined with 1 mL of 10% papaya pulp juice orally for 8 weeks, while semaglutide alone (0.9 mg/kg, s.c.) served as the pharmacological comparator. The combination improved antioxidant defenses, including GSH, SOD, CAT, and GST, while reducing oxidative stress markers such as MDA and NO. It also improved insulin‐signaling and neurotrophic pathways, attenuated neuroinflammation, and preserved neuronal architecture in the hippocampus and cerebral cortex. A particularly specific finding was normalization of butyrylcholinesterase activity only in the semaglutide‐papaya group, suggesting an additional effect on cholinergic regulation. However, because no papaya‐only group was included, these findings support a complementary role of papaya rather than establishing its independent neuroprotective efficacy (Zeweil et al. 2026). On the other hand, ripe *C. papaya* extract (50, 150, and 450 mg/kg for 14 days) showed neuroprotective effects in a rat model of focal cerebral ischemia by increasing antioxidant enzyme activities, including GSH‐Px, SOD, and CAT, while reducing lipid peroxidation, cerebral infarct volume, and escape latency (Kaewkaen 2019). These effects indicate that the extract mitigates oxidative stress, enhances cellular defense mechanisms, and supports neuronal protection against oxidative damage.

### 3.7 Cyclophosphamide‐, Acrylamide‐, and Fluoride‐Induced Oxidative Stress

Cyclophosphamide, acrylamide, and fluoride each induce oxidative stress and disrupt neuronal function through distinct but converging mechanisms (Kumar et al. 2024; Chen et al. 2018). Cyclophosphamide is metabolized into reactive intermediates such as acrolein, which generates excessive ROS, depletes antioxidant defenses (e.g., glutathione), and triggers lipid peroxidation, ultimately impairing neuronal membrane integrity and altering neurotransmitter release (Singh et al. 2014). Acrylamide readily crosses the blood–brain barrier and forms adducts with neuronal proteins, leading to mitochondrial dysfunction, increased ROS formation, and impaired axonal transport; this results in imbalances of key neurotransmitters such as dopamine, serotonin, and GABA (Zhao et al. 2022). Fluoride exposure promotes oxidative stress by enhancing ROS generation, inhibiting antioxidant enzymes (SOD, CAT, and GPX), and causing damage to neuronal lipids and DNA (Angwa et al. 2022; Miranda et al. 2021). Together, these agents disturb cellular redox balance, alter the synthesis and turnover of neurotransmitters, impair synaptic signaling, and ultimately compromise overall neuronal function. Luiz et al. (2020) showed that *C. papaya* leaf extract reduced cyclophosphamide‐induced oxidative stress by increasing GST and CAT activities and decreasing thiobarbituric acid reactive substances (TBARS) levels. Although no direct neurological outcomes were assessed, these antioxidant effects may contribute to neuroprotection by limiting oxidative damage, a key mechanism implicated in NDs (Luiz et al. 2020). These effects indicate that the leaf extract mitigates cyclophosphamide‐induced oxidative damage, may enhance antioxidant defense mechanisms. Similarly, Sadek (2012) reported that, in acrylamide‐intoxicated rats, aqueous *C. papaya* fruit extract (250 mg/kg/day for 40 days) significantly attenuated oxidative stress by decreasing MDA levels (a marker of lipid peroxidation) while enhancing endogenous antioxidant defenses, including reduced glutathione (GSH), SOD, and CAT, and concurrently increasing serum immunoglobulins (IgG and IgM), suggesting immunomodulatory support (Sadek 2012). These effects suggest that the extract helps counteract acrylamide‐induced oxidative damage by enhancing the immune response and supporting endogenous antioxidant systems, which may contribute to protection against oxidative‐stress‐mediated injury. Another study by Banala et al. (2018) reported that the leaf extract exhibits neuroprotective effects in pregnant Wistar rats (*n* = 6) exposed to fluoride‐induced behavioral changes and neurotransmitter imbalance. The extract was administered orally in drinking water at 50 and 100 mg/mL for 52 days and the extract significantly increased key neurotransmitters involved in cognitive function and mood regulation, including ACh, norepinephrine, dopamine, and serotonin. Simultaneously, it reduced glutamate, aspartate, and epinephrine levels (Banala et al. 2018). These findings indicate that the leaf extract protects neuronal function, restores neurotransmitter balance, and mitigates fluoride‐induced neurobehavioral deficits.

Overall, direct experimental studies suggest neuroprotective effects of *C. papaya* in several neuronal and animal models, including improvements in oxidative‐stress markers, antioxidant defenses, neurotransmitter‐related parameters, Aβ‐associated toxicity, neuronal integrity, and selected behavioral outcomes. Other studies conducted in non‐neuronal or systemic models demonstrate antioxidant, anti‐inflammatory, or immunomodulatory effects that may be relevant to mechanisms involved in neurodegeneration but should be considered indirect supporting evidence. Accordingly, the strength of evidence varies substantially according to the experimental model and the neurological relevance of the measured outcomes. The experimental findings are summarized in Table 2, while the proposed mechanistic pathways are illustrated in Figure 2.

**TABLE 2:** Experimental neurological and mechanistically relevant outcomes reported for *C. papaya* preparations.

Effect/model	*C. papaya* preparation	Experimental model/Sample size	Dose/Concentration and route	Measured outcomes	References
Okadaic‐acid zebrafish model	70% ethanol seed extract	Zebrafish Model, in vivo	50–100 mg/kg	At 100 mg/kg, increased time/entries in green T‐maze arm and light chamber and decreased time/entries in red arm and dark chamber	Shetty and Bharathi (2024)
Aβ aggregation/neuronal cytotoxicity	TBME fraction from methanolic dried‐leaf extract	PC12‐derived neurons and Aβ42 aggregation assay, in vitro	3.125–100 μg/mL	↑Microtubule stability; fraction was reported as non‐cytotoxic under tested conditions ↓Anti‐NGF‐induced cytotoxicity, Aβ42 aggregation	Savla et al. (2017)
Aluminum‐induced cognitive/oxidative model	Alcoholic leaf extract	Male Wistar rats, in vivo (*n* = 6)	200–400 mg/kg; orally	↑Acetylcholine, SOD, CAT, GR, cognitive function, memory performance ↓AChE activity, lipid peroxidation (MDA), oxidative stress, neuronal damage	Bindhu and Vijayalakshmi (2019)
Aluminum‐lactate biochemical model	Methanolic leaf extract	Male albino rats, in vivo (*n* = 6)	250 mg/kg; orally	↑Antioxidant status (vitamins C and E) ↓Brain AChE activity, lipid peroxidation/oxidative stress (MDA), cholinesterase activity, nitrite, TNF‐α, and ESR	Al‐Okbi et al. (2017)
	Methanolic fruit extract				
	Petroleum ether seed extract				
Nrf2/6‐OHDA cellular model	Fermented papaya preparation	Primary rat‐embryo astrocytes and dopaminergic‐neuron cell culture, in vitro (*n* = 4–6)	FPP 3 mg/mL	Increased nuclear Nrf2, NQO1, GST, HO‐1, GSH, and metallothionein‐expressing astrocytes; reduced 6‐OHDA‐induced dopaminergic neuronal loss in culture systems.	Murakami et al. (2018)
		Male ICR mice, in vivo (*n* = 8)	0.5 g/kg/day for 2 week		
D‐galactose‐induced brain‐aging model	Seed and pulp extract	Albino Wistar rats, in vivo (*n* = 5)	150 mg/kg; i.p.	↑Cognitive learning skills, memory, muscular strength, spatial memory, GABA, SOD, GPX, CAT ↓Oxidative stress, anxiety levels	Naz et al. (2022)
Systemic oxidative‐damage model (Indirect)	Fruit extract	Albino mice, in vivo (*n* = 8)	100 mg/kg; orally	↑GST, GPX, GR, G6PDH. No direct neurological outcome was reported.	Oloyede et al. (2011)
Cyclophosphamide‐induced systemic oxidative stress (Indirect)	Leaf extract	male Swiss mice, in vivo	500 mg kg‐1; Gavage	↑ GST, CAT ↓Glutathione, TBARS (lipid peroxidation), oxidative stress/damage. No direct neurological outcome was reported.	Luiz et al. (2020)
Acrylamide‐induced systemic oxidative stress (Indirect)	Fruit aqueous extract	Albino Wistar rats, in vivo (*n* = 15)	250 mg/kg; orally for 40 days	↑ GSH, SOD, CAT, IgG, IgM ↓ MDA. No direct neurobehavioral outcome was reported.	Sadek (2012)
Fluoride‐associated behavioral/neurotransmitter model	Leaf extract	Pregnant Wistar rats, in vivo (*n* = 6)	50 and 100 mg/mL through water for 52 days	↑Ach, norepinephrine, dopamine, serotonin ↓Glutamate, aspartate, epinephrine	Banala et al. (2018)
H_2_O_2_‐induced neuronal oxidative stress	Leaf extract	Human SH‐SY5Y neuroblastoma cells, in vitro	0–200 μg/mL	↑Cell viability Nrf‐2 and HO‐1 expression, BCL‐2 ↓ROS, BAX, cleaved caspase‐9, cleaved caspase‐3 and cleaved PARP, NF‐κB signaling	Jeon et al. (2025)
	Methanolic extract of papaya epicarp (peel/skin)	SH‐SY5Y neuroblastoma cells, in vitro	5–300 μg/mL	Reduced oxidative injury and improved cell‐protection/viability‐related outcomes within the in vitro model	Guizani et al. (2011)
Diabetic encephalopathy; combination intervention	Papaya pulp juice combined with semaglutide	Rat model, in vivo (*n* = 10)	Semaglutide 0.3 mg/kg s.c. + 1 mL 10% papaya pulp juice orally for 8 weeks; comparator semaglutide 0.9 mg/kg s.c.	↑Antioxidant defenses (GSH, SOD, CAT, GST), insulin signaling markers, cholinergic function, preserved hippocampal/cortical neuronal structure ↓Oxidative stress markers (MDA and NO), neuroinflammation	Zeweil et al. (2026)
Focal cerebral ischemia	Ripe papaya extract	Rat model, in vivo	50, 150 and 450 mg/kg for 14 days	↑GSH.px, SOD, CAT ↓Lipid peroxidation, brain infarct volume, escape latency	Kaewkaen (2019)
Aluminum‐induced neuronal oxidative injury	Epicarp peel extract	SH‐SY5Y cells, in vitro	30 μg/mL	↑ Neuroprotection ↓Oxidative damage	Waly et al. (2012)

*Note:* Several included studies used relatively small experimental sample sizes, particularly animal studies with approximately 5–8 subjects or observations. These findings should therefore be interpreted as preliminary and hypothesis‐generating, because small sample sizes may reduce statistical power, increase uncertainty in effect estimates, and limit the reproducibility and generalizability of the reported outcomes.

Abbreviations: ↑, increase/upregulation/stimulation; ↓, decrease/downregulation/inhibition; 6‐OHDA, 6‐hydroxydopamine; ACh, acetylcholine; AChE, acetylcholinesterase; AD, Alzheimer's disease; ALP, alkaline phosphatase; ALT, alanine aminotransferase; AST, aspartate aminotransferase; bw, body weight; CAT, catalase; CPLE, *Carica papaya* leaf extract; FPP, fermented papaya preparation; G6PDH, glucose‐6‐phosphate dehydrogenase; GABA, γ‐Aminobutyric acid; GPX, glutathione peroxidase; GR, glutathione reductase; GSH, reduced glutathione; GST, glutathione S‐transferase; HO‐1, heme oxygenase‐1; i.p., intraperitoneal; IgG, immunoglobulin G; IgM, immunoglobulin M; IROS, intracellular reactive oxygen species; MDA, malondialdehyde; *n*, number of animals; NO, nitric oxide; NQO1, NAD(P)H quinone oxidoreductase 1; Nrf2, nuclear factor erythroid 2–related factor 2; PEECPS, petroleum ether extract of *Carica papaya* seeds; ROS, reactive oxygen species; SOD, superoxide dismutase; TBARS, thiobarbituric acid‐reactive substances; TBME, tertiary butyl methyl ether; TNF‐α, tumor necrosis factor‐alpha.

## 4 Toxicity Profile of *Carica papaya*

Acute oral toxicity studies have generally reported low acute lethality for several *C. papaya* fruit and leaf preparations within the tested dose ranges. Aqueous fruit extract administered to Wistar albino rats showed no treatment‐related abnormalities, with a median lethal dose (LD_50_) of 2520 mg/kg and no significant hepatic, renal, or hematological changes (Oduola et al. 2007). Methanolic leaf extract in male Wistar rats caused no mortality up to 1500 mg/kg, though mild behavioral changes such as reduced motor activity, ataxia, depression, and slightly increased urine output were observed (Nkeiruka and Chinaka 2013). Aqueous leaf extract in Sprague–Dawley rats also produced no mortality or gross pathological lesions, with an LD_50_ greater than 2000 mg/kg b.w. (Halim et al. 2011). Similarly, there were no mortalities found when a methanolic papaya leaf extract was administered to Wistar mice in doses of up to 3200 mg/kg (Peristiowati and Puspitasari 2017). Repeated‐dose studies generally reported limited systemic toxicity under the tested conditions, although biochemical and hematological alterations occurred in some groups. A 42‐day administration of aqueous fruit extract in Wistar rats resulted in no clinical signs and no significant changes in body weight or hematological/biochemical parameters (Oduola et al. 2007). Aqueous leaf extract given for 28 days to Sprague–Dawley rats did not cause mortality; however, slight reductions in body weight and significant alterations in mean corpuscular volume (MCV), alanine aminotransferase (ALT), alkaline phosphatase (ALP), and triglycerides were recorded at higher doses in males (Afzan et al. 2012). A study reported that oral administration of 10% ethanolic *C. papaya* leaf extract produced no acute toxicity at 5000 mg/kg or significant chronic toxic effects at doses up to 5000 mg/kg/day for 180 days (Taychaworaditsakul et al. 2024). Overall, the studies reported low acute lethality for several tested preparations. However, behavioral, biochemical, hematological, and histopathological alterations were observed under some experimental conditions.

Overall, the available acute, repeated‐dose, and chronic animal studies suggest relatively low acute toxicity for several *C. papaya* fruit and leaf preparations within the tested experimental conditions, with no mortality reported in the cited studies. However, behavioral alterations, changes in biochemical and hematological parameters, and mild hepatic histopathological findings were observed in some studies, particularly at higher doses. These findings indicate that the safety profile may depend on the plant part, extraction method, administered dose, duration of exposure, and experimental model. Therefore, the available preclinical evidence should not be interpreted as establishing the long‐term safety of all *C. papaya* preparations, particularly for therapeutic use in humans. Further standardized long‐term toxicity and clinical safety studies are required.

## 5 Human Evidence and Its Relevance to Neuroprotection

Human studies of *C. papaya* preparations have primarily investigated non‐neurological conditions and therefore provide information on human exposure, tolerability, and systemic biological activity rather than direct evidence of neuroprotection. Kasture et al. (2016) reported that *C. papaya* leaf extract accelerated platelet recovery in patients with dengue‐associated thrombocytopenia and was generally well tolerated (Kasture et al. 2016). Similarly, Gadhwal et al. (2016) reported increased platelet counts and reduced platelet‐transfusion requirements following *C. papaya* leaf extract administration in patients with dengue‐associated thrombocytopenia (Gadhwal et al. 2016). These studies provide limited information on clinical tolerability and pharmacodynamic effects of specific *C. papaya* preparations in the studied populations but do not directly support efficacy against NDs. Further research by Kharaeva et al. (2022) evaluated a combined fermented *C. papaya* and *Morinda citrifolia* preparation in individuals with post‐COVID symptoms and reported changes in systemic inflammatory and oxidative‐stress‐related markers, including IL‐6, IL‐8, NO metabolites, antioxidant activity, and ATP levels (Kharaeva et al. 2022). Because the intervention contained more than one botanical preparation and was not designed to assess a ND or validated neurological outcomes, these findings should be regarded only as indirect evidence of systemic antioxidant and immunomodulatory activity and should not be interpreted as direct support for the neuroprotective efficacy of *C. papaya* . Further research demonstrated that supplementation with fermented *C. papaya* and *Morinda citrifolia* significantly improved post‐COVID symptoms compared with placebo. The intervention reduced circulating IL‐6 and IL‐8 and NO metabolites, while enhancing plasma antioxidant activity, polymorphonuclear leukocyte phagocytic capacity, and ATP levels. Improvements in cardiac and pulmonary function and overall physical well‐being were also observed (Kharaeva et al. 2022). However, no eligible clinical study identified in this review evaluated *C. papaya* for a diagnosed neurodegenerative disorder. Overall, currently available human studies do not provide direct clinical evidence that *C. papaya* prevents or treats NDs. The existing non‐neurological clinical studies may provide supportive information regarding tolerability and systemic biological effects, but neurological efficacy requires dedicated clinical trials using standardized *C. papaya* preparations and validated neurological outcomes.

## 6 Synthesis of Evidence and Knowledge Gaps

The available evidence comprises both direct neuroprotective studies and indirect mechanistic studies. Direct evidence arises from neuronal cell systems and animal models in which neuronal, neurochemical, neuropathological, cognitive, or behavioral outcomes were assessed, including selected models of Alzheimer's‐like pathology, aluminum‐induced neurotoxicity, D‐galactose‐induced brain aging, 6‐OHDA‐induced dopaminergic injury, fluoride‐associated neurobehavioral alterations, and cerebral ischemia. In contrast, studies examining systemic antioxidant activity, endothelial oxidative stress, xanthine oxidase inhibition, cyclophosphamide‐ or acrylamide‐associated systemic oxidative stress, dengue thrombocytopenia, and post‐COVID outcomes provide indirect evidence only. Although these studies identify antioxidant, anti‐inflammatory, or immunomodulatory activities that may be mechanistically relevant to neuronal injury, they do not independently demonstrate neuroprotection.

Synthesizing evidence from peer‐reviewed studies, this review indicates that *C. papaya* preparations show neuroprotective effects in several experimental neurological models, including Alzheimer's disease, aluminum‐induced neurotoxicity, D‐galactose‐induced brain aging, 6‐OHDA‐induced dopaminergic injury, and fluoride‐associated neurobehavioral alterations. These effects are associated with reductions in oxidative stress markers, including MDA and TBARS, restoration of endogenous antioxidant defenses such as SOD, CAT, GPX, and GR, attenuation of Aβ aggregation, and modulation of neurotransmitters including ACh, dopamine, serotonin, and GABA. Evidence from cyclophosphamide‐ and acrylamide‐induced oxidative stress models is indirect because neurological endpoints were not assessed; however, the reported antioxidant effects may be mechanistically relevant to neuroprotection. Mechanistically, FPP has been reported to activate Nrf2‐mediated antioxidant signaling and increase downstream cytoprotective molecules, including HO‐1, NQO1, and GST, whereas leaf extracts have shown AChE inhibitory activity and modulation of monoamine levels in selected experimental models. Overall, these findings suggest that *C. papaya* may act through multiple mechanisms relevant to neuronal protection, although the strength and directness of the evidence vary across experimental systems.

However, despite this multiple preclinical evidence, critical knowledge gaps persist: (i) the specific bioactive compounds responsible for these effects remain unidentified, as most studies utilize crude extracts; (ii) pharmacokinetic data on bioavailability and blood–brain barrier penetration were not identified among the included studies; (iii) standardized extraction protocols and validated dose–response relationships remain undefined; (iv) several studies employed small experimental sample sizes, including groups of approximately 5–8 animals, which may reduce statistical power and increase uncertainty around reported effect sizes; and (v) no eligible clinical trials evaluating neurological efficacy in humans were identified in this review. Therefore, future investigations must prioritize bioassay‐guided fractionation to pinpoint active constituents, comprehensive pharmacokinetic profiling, and rigorously designed randomized controlled trials to determine whether the reported preclinical findings have translational relevance to human NDs.

## 7 Novelty and Future Perspectives

This review provides a comparative evaluation of *C. papaya* 's neuroprotective potential across diverse experimental models of neurodegeneration, including AD, aluminum‐induced toxicity, D‐galactose‐induced aging, and chemically induced oxidative stress. A strength of this review is its integration of findings from seeds, leaves, fruits, fermented preparations, and different extract types, highlighting a broad spectrum of neuroprotective actions such as antioxidant enhancement, neurotransmitter restoration, anti‐inflammatory activity, and suppression of Aβ aggregation. By consolidating evidence from multiple in vitro and in vivo studies, the available evidence suggests that selected *C. papaya* preparations warrant further investigation for mechanisms potentially relevant to NDs.

Future research should aim to isolate the key bioactive compounds responsible for *C. papaya* 's neuroprotective effects and clarify their molecular mechanisms using advanced biochemical and molecular tools. Standardized dosing, pharmacokinetic studies, and long‐term safety evaluations are needed to establish therapeutic relevance. Importantly, well‐designed clinical trials are required to evaluate its efficacy and safety in humans. Further exploration of nanoparticle delivery systems, synergistic combinations with other phytochemicals, and optimized formulations could be investigated after the active constituents, pharmacokinetics, and safety profiles are better characterized for multi‐target natural therapy for ND diseases.

## 8 Conclusion

The evidence compiled in this review suggests potential neuroprotective effects of selected *C. papaya* preparations in preclinical experimental models involving oxidative stress, neuroinflammation, neurotransmitter imbalance, and Aβ aggregation. Studies of extracts prepared from different plant parts reported improvements in selected cognitive, behavioral, biochemical, and neuronal outcomes, although the findings varied by preparation and experimental model. However, available evidence is predominantly preclinical. In neuronal‐cell and animal studies, *C. papaya* preparations were associated with changes in measured outcomes including oxidative‐stress markers, antioxidant‐enzyme activities, cholinesterase and neurotransmitter‐related measures, Aβ‐associated cellular toxicity or aggregation, neuronal viability or histology, cerebral infarct volume, and selected behavioral tests. The findings varied by preparation and model, and several studies used small experimental groups. Studies using systemic or non‐neuronal models provide indirect mechanistic support rather than direct evidence of neuroprotection. Toxicity studies reported no mortality at several tested doses but also described behavioral, biochemical, hematological, or histopathological changes under some conditions. No clinical trial identified in this review tested *C. papaya* for a diagnosed neurodegenerative disorder. The current evidence therefore supports further experimental investigation but does not establish clinical efficacy, an optimal dose, or long‐term neurological safety in humans.

## Author Contributions

**Rakib Hossan:** conceptualization, writing – original draft, writing – review and editing, formal analysis, software, validation, resources, data curation. **Samy Selim:** conceptualization, writing – original draft, writing – review and editing, visualization, validation, methodology, software, project administration, supervision, resources, formal analysis. **Imam Hossen Rakib:** conceptualization, writing – original draft, writing – review and editing. **Md Abu Sayeed:** conceptualization, writing – original draft, writing – review and editing. **Anike Chakrabarty:** writing – original draft, writing – review and editing, validation, investigation, conceptualization, software, formal analysis. **Md. Arif Hossain:** validation, methodology, investigation, software, visualization. **Emon Mia:** writing – original draft, conceptualization, writing – review and editing, formal analysis, software. **Dristy Debnath:** investigation, visualization, software. **Asmaul Husna Bristy:** investigation, visualization, methodology, software, resources. **Md. Sakib Al Hasan:** conceptualization, writing – original draft, writing – review and editing, visualization, formal analysis, project administration, supervision, software.

## Funding

The authors have nothing to report.

## Disclosure

Copyright/Permission Statement: The authors confirm that all tables were prepared using data extracted and synthesized from previously published studies, with appropriate citations and references provided. All figures were originally designed and created by the authors using Microsoft PowerPoint based on the reviewed literature and do not reproduce previously published figures.

Author Responsibility Statement: All authors have read and approved the final version of the manuscript. Dr. Samy Selim and Md. Sakib Al Hasan, as corresponding authors, had full access to the source materials and extracted evidence used in this review and take responsibility for the integrity and accuracy of the evidence synthesis.

## Conflicts of Interest

The authors declare no conflicts of interest.

## Data Availability

No new data were generated or analyzed in this review article. All information discussed in the manuscript was derived from previously published and publicly available literature, which has been appropriately cited and referenced.
